# The *velvet* Regulator VosA Governs Survival and Secondary Metabolism of Sexual Spores in *Aspergillus nidulans*

**DOI:** 10.3390/genes11010103

**Published:** 2020-01-16

**Authors:** Min-Ju Kim, Mi-Kyung Lee, Huy Quang Pham, Myeong Ju Gu, Bohan Zhu, Sung-Hun Son, Dongyup Hahn, Jae-Ho Shin, Jae-Hyuk Yu, Hee-Soo Park, Kap-Hoon Han

**Affiliations:** 1 School of Food Science and Biotechnology, Kyungpook National University, Daegu 41566, Korea; 13mjkim@gmail.com (M.-J.K.); myungju1260@naver.com (M.J.G.); rk05555@naver.com (S.-H.S.); dohahn@knu.ac.kr (D.H.); 2 Biological Resource Center (BRC), Korea Research Institute of Bioscience and Biotechnology (KRIBB), Jeongeup-si 56212, Korea; miklee1010@kribb.re.kr; 3 School of Applied Biosciences, Kyungpook National University, Daegu 41566, Korea; bongbimit@gmail.com (H.Q.P.); jhshin@knu.ac.kr (J.-H.S.); 4 Department of Pharmaceutical Engineering, Woosuk University, Wanju 55338, Korea; allen9055@163.com; 5 Department of Integrative Biology, Kyungpook National University, Daegu 41566, Korea; 6 Departments of Bacteriology and Genetics, University of Wisconsin, Madison, WI 53706, USA; jyu1@wisc.edu; 7 Department of Systems Biotechnology, Konkuk University, Seoul 05029, Korea

**Keywords:** VosA, sexual development, ascospores, *Aspergillus nidulans*

## Abstract

The *velvet* regulator VosA plays a pivotal role in asexual sporulation in the model filamentous fungus *Aspergillus nidulans*. In the present study, we characterize the roles of VosA in sexual spores (ascospores) in *A*. *nidulans*. During ascospore maturation, the deletion of *vosA* causes a rapid decrease in spore viability. The absence of *vosA* also results in a lack of trehalose biogenesis and decreased tolerance of ascospores to thermal and oxidative stresses. RNA-seq-based genome-wide expression analysis demonstrated that the loss of *vosA* leads to elevated expression of sterigmatocystin (ST) biosynthetic genes and a slight increase in ST production in ascospores. Moreover, the deletion of *vosA* causes upregulation of additional gene clusters associated with the biosynthesis of other secondary metabolites, including asperthecin, microperfuranone, and monodictyphenone. On the other hand, the lack of *vosA* results in the downregulation of various genes involved in primary metabolism. In addition, *vosA* deletion alters mRNA levels of genes associated with the cell wall integrity and trehalose biosynthesis. Overall, these results demonstrate that the *velvet* regulator VosA plays a key role in the maturation and the cellular and metabolic integrity of sexual spores in *A. nidulans*.

## 1. Introduction

*Aspergillus* species are one of the most ubiquitous fungi and inhabit a variety of environmental niches [1]. To propagate under diverse environmental conditions, these fungi produce two types of spores, conidia (asexual spores) and ascospores (sexual spores) [2,3,4]. Most *Aspergillus* species reproduce asexually by forming specialized developmental structures: Conidiophores bearing conidia [5]. Airborne conidia are small (less than 10 μm) and light; therefore, they can disperse easily or be inhaled by humans, thereby causing disease in an immunocompromised host [6]. Some members of the genera *Aspergillus* can also undergo sexual cycles and produce sexual structures [7]. For example, the species of *Aspergillus* section *Nidulantes* produce sexual fruiting bodies called cleistothecia [8]. Several species of *Aspergillus* section *Flavi* produce sclerotia [9]. The morphologies of asexual and sexual structures are important species-specific characteristics, thus morphology is used for taxonomy [10]. The processes of sexual and asexual structure formation are complicated and are tightly regulated by a variety of regulators [4,11]. These studies have primarily focused on the model fungus *Aspergillus nidulans* [12,13].

*A*. *nidulans* is the model organism for genetic studies on fungal biology [12,14]. *A*. *nidulans* is a homothallic fungus that can produce sexual fruiting bodies without mating partners [7,15]. Under the appropriate conditions, *A*. *nidulans* hyphae undergo sexual cycles and form coiled lumps, ascogenous hyphae, and cleistothecia in sequence [16]. During the development of cleistothecia, the Hülle cells surround young cleistothecium and support their maturation [16]. Each mature cleistothecium contains approximately 10,000 ascospores [17]. During sexual cycles, various genes are associated with the formation and maturation of cleistothecium [7]. For example, NsdC and NsdD are essential for the initiation of sexual development [18,19,20]. The deletion of *nsdC* or *nsdD* leads to the inability to form sexual fruiting bodies [18,20]. 

The velvet family of proteins is comprised of fungus-specific transcription factors that govern asexual/sexual reproduction and secondary metabolite biosynthesis [21]. These transcription factors can form various types of complexes that play distinct roles in *A*. *nidulans* [22]. The VelB-VeA-LaeA complex regulates the initiation of sexual reproduction, Hülle cell formation, and secondary metabolite biosynthesis [23]. VosA-VelB, another velvet complex, plays a crucial role in spore viability and the maturation of asexual spores [24,25]. Deletion of *vosA* or *velB* decreases asexual spore viability and trehalose contents in conidia [24]. Previous studies have demonstrated that VosA contains a velvet domain that binds to the promoter regions of genes associated with spore maturation and developmental processes [26,27]. Microarray results revealed that VosA regulates mRNA expression of genes involved in cell wall integrity in conidia [27]. The function of VosA in asexual spores has been studied in other *Aspergillus* species and is conserved in trehalose biosynthesis and stress response in asexual spores [28,29]. The role of VosA in asexual spores is well described; however, its role in sexual spores is thus far not well understood. In this study, we investigated the role of VosA in sexual spores in depth. To test whether VosA plays a similar role in ascospores, we conducted phenotypic, metabolomic, and genomic analyses in *A*. *nidulans* ascospores. This study elucidates the roles of VosA in developmental and metabolic processes during the sexual reproduction of *A*. *nidulans*.

## 2. Materials and Methods

### 2.1. Strains, Media, and Culture Conditions

Wild type (WT, FGSC 4), *vosA* deletion mutant (Δ*vosA*, THS15.1), and *vosA* complemented (C’ *vosA*, THS28.1) strains were used in this study [24,27]. All fungal strains were grown on solid minimal medium (MM) or sexual media (SM) with supplements, as previously described [30].

### 2.2. Ascospore Viability

To determine the viability of ascospores in cleistothecium, WT and mutant strains were inoculated onto SM and incubated at 37 °C for 7 or 14 days. After incubation, 10 individual cleistothecia were isolated from the plates, washed with ddH_2_O to remove conidia, and transferred to a new tube in 100 μL of ddH_2_O. The number of ascospores was counted in a hemocytometer. After dilution, approximately 100 ascospores were plated onto MM agar plates and incubated at 37 °C for 2 days. The numbers of colonies were then counted. All experiments were carried out in triplicate.

### 2.3. Ascospore Trehalose Assay

The trehalose assay was performed as previously described with modification [24]. WT and mutant strains were grown with SM at 37 °C for 7 days in the dark. After 7 days, cleistothecia were collected from plates, washed using ddH_2_O to remove hyphae or conidiophores, and broken with a dounce homogenizer. Broken cleistothecia were passed through Miracloth to collect pure ascospores. Ascospore suspensions (10^8^) were suspended in 200 μL of ddH_2_O and incubated for 20 min at 95 °C. After centrifugation, the supernatant was mixed with 0.2 M sodium citrate (pH 5.5) and incubated with or without trehalase (Sigma, St. Louis, MO, USA) for 8 h at 37 °C. Trehalose is converted to glucose by trehalase. After incubation, the amount of glucose was assayed with a glucose assay kit (Sigma, St. Louis, MO, USA) following the manufacturer’s instruction. All experiments were carried out in triplicate.

### 2.4. Ascospore Stress Tolerance Test

Stress tolerance tests were carried out as described previously [31]. WT and mutant ascospores were collected from 7-day cultures on SM plates, as described above. For oxidative tolerance tests, ascospore suspensions containing 10^5^ ascospores were incubated with various concentrations of H_2_O_2_ for 30 min. For thermal tolerance tests, 10^5^ ascospore suspensions were incubated at 50 °C for 0, 15, and 30 min. After incubation under oxidative or thermal stress conditions, ascospore suspensions were diluted with ddH_2_O. The diluted ascospores were inoculated onto solid MM and incubated at 37 °C for 2 days. Colony numbers were counted and calculated as the ratio of viable colonies relative to untreated controls. All experiments were carried out in triplicate.

### 2.5. Sterigmatocystin (ST) Extraction and HPLC Conditions

To extract sterigmatocystin from ascospores, 10^9^ ascospores from 7-day cultures of cleistothecia were extracted by adding 2 mL of CHCl_3_. The organic phase was separated by centrifugation and transferred to new vials. After transfer, the organic phase was evaporated and resuspended in 0.5 mL HPLC-grade acetonitrile:methanol (50:50, *v*/*v*). The samples were passed through a 0.45 μm pore filter.

Analytical reverse-phase high-performance liquid chromatography (RP-HPLC) was conducted on a Waters 2695 (separation module) with a Waters 2996 photodiode array detector (Waters, Milford, MA, USA) and a quaternary pump, using a HECTOR-M-C18 column (5 µm × 4.6 mm × 250 mm, RS tech corporation, Daejeon, Korea). A sample volume of 10 μL was injected into the column. The mobile phase consisted of acetonitrile:water (60:40, *v*/*v*). The flow rate was 0.8 mL/min. A sterigmatocystin stock solution (Sigma, St. Louis, MO, USA) was dissolved in acetonitrile:methanol (50:50, *v*/*v*). A linear calibration curve (*R*^2^ = 0.999) was constructed with various concentrations of sterigmatocystin. Sterigmatocystin was detected at a wavelength of 246 nm. The retention time for sterigmatocystin was approximately 9.7 min.

### 2.6. RNA Sequencing (RNA-seq)

To isolate the total RNA for RNA-seq experiments, triplicate samples of conidia from WT and mutant strains were inoculated onto solid MM and incubated at 37 °C for 7 days in the dark. Cleistothecia were collected from the 7-day cultures, washed using ddH_2_O, broken with a Dounce homogenizer, and passed through Miracloth (Calbiochem, San Diego, CA, USA). Afterward, pure ascospores were collected for total RNA extraction. Total RNA from WT and mutant ascospores was extracted using Trizol reagent (Invitrogen, Waltham, MA, USA) according to the manufacturer’s instructions with modification. RNA samples were submitted to the University of Wisconsin Gene Expression Center (Madison, WI, USA) for library preparation and sequencing. A strand-specific library was prepared using an Illumina TruSeq strand-specific RNA sample preparation system. The libraries of all the replicates were sequenced using an Illumina HiSeq 2500 system. 

### 2.7. Data Analysis

Low-quality reads were identified by the following criteria: Reads containing more than 10% of skipped bases (marked as ‘N’s), reads containing more than 40% of bases whose quality scores were less than 20 and reads whose average quality score for each read was less than 20. The filtering process was performed using the in-house scripts. Filtered reads were mapped to the *A*. *nidulans* A4 transcriptome using the aligner STAR v.2.3.0e [32]. Gene expression levels were measured with Cufflinks v2.1.1 [33] using the gene annotation database from the *Aspergillus* Genome Database (AspGD) [34]. To improve accuracy, multi-read-correction and fragbias-correct options were applied. All other options were set to default values. For differential expression analysis, gene-level count data were generated using HTSeq-count v0.5.4p3 [35] with the options “-m intersection-nonempty” and “–r option considering paired-end sequence.” Based on the calculated read count data, differentially expressed genes (DEGs) were identified using the R package called TCC [36]. The TCC package applied robust normalization strategies to compare tag count data. Normalization factors were calculated using the iterative DEGES/edgeR method. The *q*-value was calculated based on the *p*-value using the *p*-adjust function of the R package with default parameter settings. DEGs were identified based on a *q*-value threshold of less than 0.05. RNA-seq sequence data are available through the NCBI SRA database under the BioProject accession number PRJNA588808.

### 2.8. Gene Ontology Enrichment Analyses

Gene ontology enrichment analyses were carried out using the tools available at the AspGD (AspGD Gene Ontology Slim Mapper, the process category) [34] and FungiFun database [37]. To characterize the genes identified from DEG analysis, a GO-based trend test was performed using Fisher’s exact test; *p*-values < 0.001 were considered statistically significant.

### 2.9. Real-Time PCR Analysis

For real-time PCR analysis, total RNA was isolated as described above. Complementary DNA (cDNA) was synthesized using the GoScript Reverse Transcription system (Promega, Madison, WI, USA). Quantitative real-time PCR was performed with each gene-specific primer set and iTaq universal SYBR Green supermix (Bio-Rad, Hercules, CA, USA) using a CFX96 Touch Real-Time PCR system (Bio-Rad). To calculate the expression levels of target genes, the 2^−∆∆CT^ method was used. The expression of β-actin was used as an endogenous control. All experiments were carried out in triplicate. The oligonucleotides used in these experiments are listed in Table 1. 

### 2.10. Statistical Analysis

Statistical differences between WT and mutant strains were evaluated by the Student’s unpaired *t*-test. Mean ± standard deviation (SD) are shown. *p*-Values < 0.05 were considered statistically significant.

## 3. Results

### 3.1. The VosA Gene is Required in Ascospore Viability 

**Figure 1:**
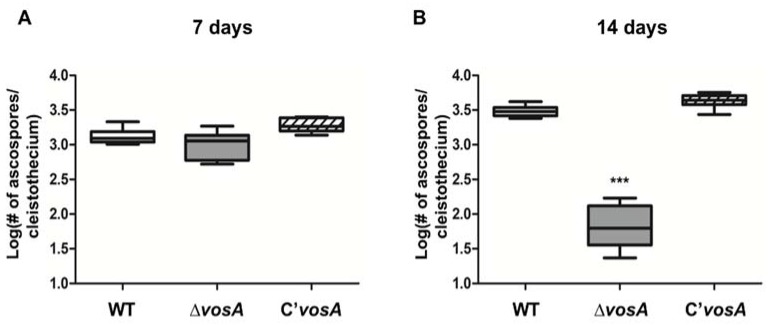
Viability of ascospores of the Δ*vosA* mutant. (**A**,**B**) The number of colony-forming ascospores (log scale) per cleistothecium of wild type (WT) (FGSC 4), Δ*vosA* (THS15.1), and C’*vosA* (THS28.1) strains. Ten independent cleistothecium of WT were collected from plates cultured for 7 (**A**) or 14 (**B**) days. Ascospores from each cleistothecium were collected, spread onto MM solid media, and incubated for 48 h. Ascospore viability was estimated by counting the colony-forming units (WT vs. Δ*vosA*, *** *p* < 0.001).

A previous study found that *vosA* was essential for spore viability [25]. Deletion of *vosA* resulted in the lack of conidia viability. In addition, ascospores from 24 days cultures of Δ*vosA* lack cytoplasm and organelles. To further assess the role of *vosA* in ascospores, we examined cleistothecia and ascospores from WT and mutant strains. The size and distribution of Δ*vosA* cleistothecia were similar to those of WT cleistothecia. WT and Δ*vosA* cleistothecia collected after 7 and 14 days of incubation contained 10^5^–10^6^ ascospores. Among them, between 1000 and 2000 ascospores collected after 7 days incubation were capable of colony formation in all strains (Figure 1). Ascospores from the cleistothecia of mature WT and complemented strains isolated after 14 days incubation formed 2500–5000 colonies, whereas ascospores from mature Δ*vosA* cleistothecia formed less than 100 colonies. These results indicate that VosA is crucial for ascospore viability during the maturation process. 

### 3.2. Genome-Wide Analysis Reveals That VosA Affects Secondary Metabolism Gene Expression in Ascospores

**Figure 2:**
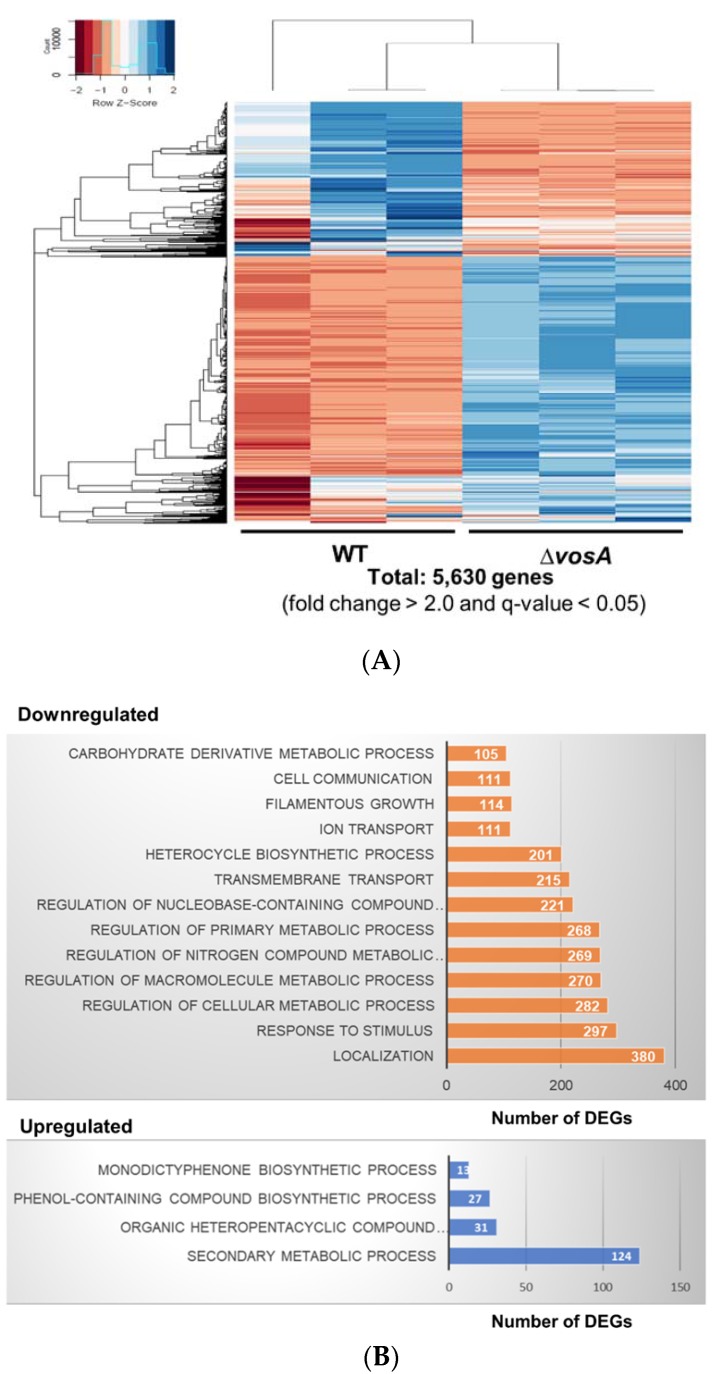
Genome-wide analyses of genes whose expression is affected by VosA in ascospores. (**A**) Hierarchical clustering analysis of differentially expressed genes (DEGs) between WT and Δ*vosA* ascospores (fold change > 2.0 and *q*-value < 0.05) is presented. (**B**) Gene Ontology (GO) term enrichment analysis of DEGs in the Δ*vosA* ascospores.

To gain insight into the regulatory role of VosA in ascospores, we performed RNA-seq experiments. The analysis of the results shows that there were 5630 DEGs between WT and Δ*vosA* ascospores from 7 days old cleistothecia (Figure 2, fold change >2.0 for upregulation or downregulation, and *q*-value < 0.05). The mRNA expression levels of 2334 genes were downregulated in Δ*vosA* ascospores compared with WT ascospores. However, loss of *vosA* led to the upregulation of the mRNA expression of 3296 genes in ascospores. To further elucidate the role of VosA, functional enrichment analyses were carried out (Supplementary Tables S1 and S2). GO analyses demonstrated that a large number of genes involved in the biosynthesis of secondary metabolites, monodictyphenones, and organic heteropentacyclic compounds were differentially upregulated in Δ*vosA* ascospores. However, genes involved in regulating primary metabolism, responding to stimuli, and macromolecule metabolism, were downregulated in Δ*vosA* ascospores. These results indicate that VosA induces the expression of genes that have roles in primary metabolic processes or that repress the expression of genes involved in secondary metabolic processes. 

### 3.3. VosA Regulates Trehalose Biosynthesis in Ascospores

**Figure 3:**
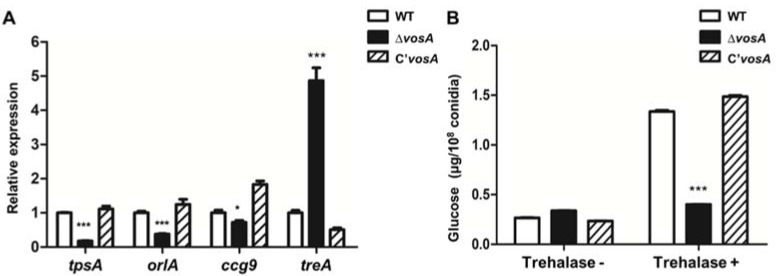
Trehalose biosynthesis in Δ*vosA* ascospores. (**A**) Levels of *tpsA*, *orlA*, *ccg9*, and *treA* mRNA in WT (FGSC 4), Δ*vosA* (THS15.1), and C’*vosA* (THS28.1) ascospores (WT vs. Δ*vosA*, *** *p* < 0.001). (**B**) The amount of glucose (μg) per 10^8^ ascospores from 7 days cultures of cleistothecia from WT (FGSC 4), Δ*vosA* (THS15.1), and C’*vosA* (THS28.1) strains (measured in triplicate). Trehalose is converted to D-glucose by trehalase. The amount of glucose was assayed with a glucose assay kit. Untreated trehalase samples were used as negative controls (WT vs. Δ*vosA*, * *p* < 0.05; *** *p* < 0.001).

VosA is a key regulator of trehalose biosynthesis in *Aspergillus* asexual spores [25,28,29]. Transcriptomic analysis data suggested that the absence of *vosA* affected expression of genes associated with trehalose biosynthesis. Levels of *tpsA* (AN5523), *orlA* (AN3441), and *ccg9* (AN5021), which are involved in trehalose synthesis, were lower in Δ*vosA* ascospores compared to WT ascospores. Levels of *treA*, which is also involved in trehalose hydrolysis, were higher in Δ*vosA* ascospores. We verified *tpsA*, *orlA*, *ccg9*, and *treA* mRNA levels using qRT-PCR (Figure 3A). To assess whether the altered expression of these genes affected biological processes, the trehalose content in WT and mutant ascospores was measured. As shown in Figure 3B, the amount of trehalose in Δ*vosA* ascospores was significantly less than in ascospores from WT and complemented strains. These results show that VosA controls (directly or indirectly) the expression of genes with important roles in the synthesis of trehalose in *A*. *nidulans* ascospores. 

### 3.4. Deletion of vosA Alters the Oxidative and Thermal Stress Response 

**Figure 4:**
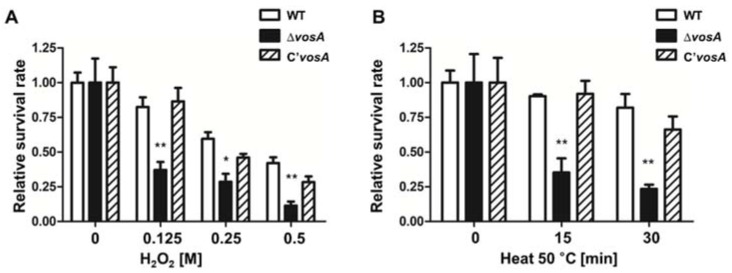
Role of VosA in stress tolerance of ascospores. (**A**,**B**) Tolerance of WT (FGSC 4), Δ*vosA* (THS15.1), and C’*vosA* (THS28.1) ascospores to oxidative (**A**) and thermal (**B**) stresses (WT vs. Δ*vosA*, * *p* < 0.05; ** *p* < 0.01).

Because trehalose is a key protectant in various environmental responses, a lack of trehalose can reduce stress tolerance [38,39]. The deletion of *vosA* results in decreased trehalose content in ascospores, implying that Δ*vosA* ascospores might be more sensitive to environmental stresses. To test this hypothesis, we collected ascospores from 7 days cleistothecia and examined oxidative and thermal stress tolerance. In response to both stresses, Δ*vosA* ascospores were more sensitive compared with WT and complemented strain ascospores (Figure 4). Taken together, these results suggested that VosA was required for proper oxidative and thermal stress tolerance in ascospores. 

### 3.5. VosA Is Involved in the Production of Secondary Metabolites 

**Figure 5:**
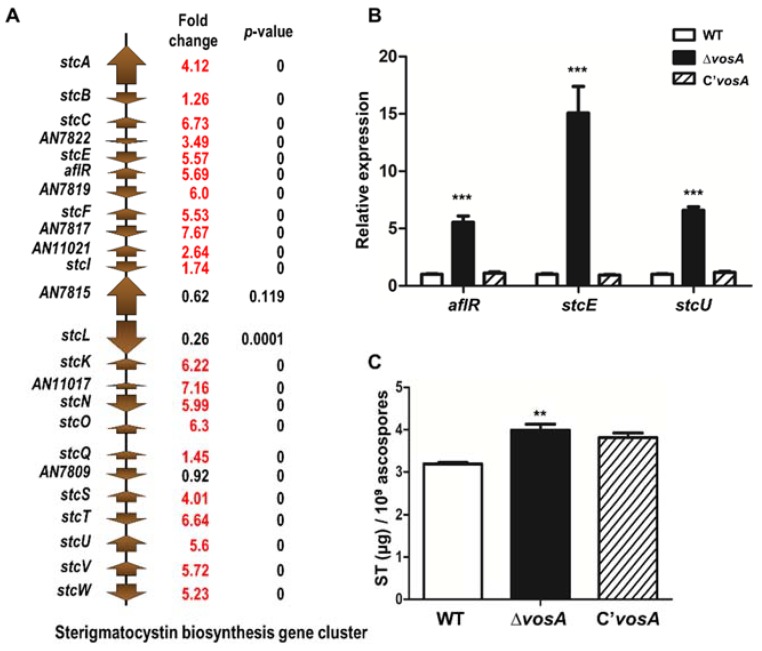
Sterigmatocystin production in the Δ*vosA* mutant ascospores. (**A**) Differentially regulated genes involved in the sterigmatocystin gene clusters in the Δ*vosA* ascospores. (**B**) mRNA expression of *aflR*, *stcE*, and *stcU* in WT, Δ*vosA*, and C’*vosA* ascospores. (**C**) Sterigmatocystin was extracted from 10^9^ ascospores of WT, Δ*vosA*, and C’*vosA* strains. Extracted sterigmatocystin was measured by high-performance liquid chromatography (HPLC) (measured in triplicate). (WT vs. Δ*vosA*, ** *p* < 0.01, *** *p* < 0.001).

As mentioned above, Δ*vosA* ascospores exhibited altered mRNA expression of genes associated with secondary metabolites by transcriptomic analysis. We screened mRNA levels of DEGs from 25 secondary metabolite gene clusters in the RNA-seq data (Supplementary Table S3). Among them, many genes in 7 secondary metabolites gene clusters were upregulated in Δ*vosA* ascospores (Table 2). These gene clusters were asperthecin, microperfuranone, monodictyphenone, sterigmatocystin, *pkdA*, and derivative of benzaldehyde and F9775 hybrid clusters 1 and 2. For the sterigmatocystin gene cluster, 21 of the 24 genes, including *aflR*, *stcE*, and *stcU*, were upregulated in Δ*vosA* ascospores (Figure 5A). Only 3 genes (AN7809, AN7814, AN7815) were not upregulated in Δ*vosA* ascospores. qRT-PCR analysis verified that *aflR*, *stcE*, and *stcU* mRNA levels in Δ*vosA* ascospores were higher than in WT ascospores (Figure 5B). Because the expression of genes in the sterigmatocystin cluster was increased, we measured sterigmatocystin production in WT and mutant ascospores. As shown in Figure 5C, sterigmatocystin production in Δ*vosA* ascospores was slightly increased compared with that in WT ascospores, but there was no significant difference in sterigmatocystin production between Δ*vosA* ascospores and complemented strain ascospores. These results suggested that VosA was required for the regulation of mRNA expression of secondary metabolite gene clusters.

## 4. Discussion

Previous transcriptomic analysis demonstrated that VosA regulates mRNA expression of cell wall-related genes and secondary metabolite cluster genes in conidia [27]. Absence of *vosA* results in decreased mRNA expression of genes associated with trehalose biosynthesis, but mRNA levels of genes involved in β-glucan biosynthesis and chitin biosynthesis in asexual spores are increased [27]. Our RNA-seq results also found that VosA is involved in transcriptional regulation of cell wall-related genes and secondary metabolite cluster genes. To understand the role of VosA in both asexual and sexual spores, we compared microarray data of Δ*vosA* conidia and RNA-seq data of Δ*vosA* ascospores. As shown in Supplementary Figure S1, 464, and 588 genes in ∆*vosA* conidia and ascospores were upregulated and downregulated, respectively. We also carried out GO analysis using overlapped genes. Expression of secondary metabolism-related genes was increased in both asexual and sexual spores. Interestingly, the expression of genes involved in sterigmatocystin biosynthetic process is also upregulated in Δ*vosA* ascospores. One possible explanation is that the absence of VosA increases free VelB, facilitating the formation of a VelB–VeA–LaeA complex [23], a key regulator of sterigmatocystin biosynthesis. Further investigation of the dynamics of velvet complex formation will provide insight into the molecular mechanisms of sterigmatocystin biosynthesis. In both spores, mRNAs of genes associated with asexual developmental processes (*brlA*, *abaA*, *stuA*, *sltA*) and septum processes (*cdcA*, *aspC*, *aspD*, *sidB*) were upregulated. Although the expression of developmental genes should be repressed during spore maturation and dormancy, absence of *vosA* altered mRNA expression of these genes, which could affect the phenotype of spores. 

Because trehalose protects *Aspergillus* species from environmental stresses, genes involved in trehalose biosynthesis are considered as potential targets for antifungal drug development [39,40]. In Δ*vosA* conidia and ascospores, trehalose content was dramatically decreased compared with WT spores. In addition, mRNA levels of trehalose biosynthesis-related genes (*tpsA* and *orlA*) were decreased in both spores, suggesting that VosA affects trehalose biosynthesis by regulating transcription of trehalose biosynthesis-related genes.

Previous work found that the VosA–VelB complex represses the germination of asexual spores [24]. The deletion of *vosA* accelerates the germination rate of asexual spores in *A*. *nidulans* [24]. The role of VosA in conidial germination is similar in other *Aspergillus* species, such as *A*. *fumigatus*. We speculate that VosA could play a similar role in the germination of *A*. *fumigatus* ascospores. However, the estimation of ascospore germination rates is difficult because the germination activity of ascospores from 7-day cleistothecium is only 10–30% in the presence of glucose. Therefore, we checked the germination activity of ascospores in the absence of glucose. Several ascospores of Δ*vosA* mutants start to form a germ tube, but not WT and complemented strains (Supplementary Figure S2). This result is consistent with the results from studies in *A*. *nidulans* and *A*. *fumigatus* [24,29]. These results support the idea that VosA negatively regulates the germination activity of asexual and sexual spores in *Aspergillus* species. 

Although we demonstrated that VosA is a key factor for sterigmatocystin production in sexual spores by transcriptomic and sterigmatocystin production analyses, this study has limitations. The expression of many genes in the sterigmatocystin gene cluster was increased, but the slight increase in sterigmatocystin production was unexpected. We speculate that regulation at the post-transcriptional, translational, or metabolic levels are involved; however, the reason for the result observed in this study is not clear at present. Therefore, further study is needed to explain it.

**Figure 6:**
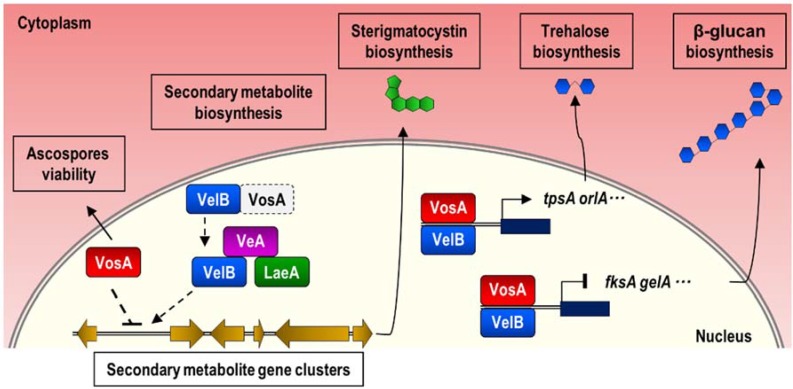
Proposed model depicting the role of VosA during ascosporogenesis. In ascospores, VosA plays a variety of roles during ascosporogenesis. First, VosA is required for ascospores viability. Second, VosA directly or indirectly controls mRNA expression of secondary metabolite gene clusters. Deletion of *vosA* causes an increase in the number of VelB–VeA–LaeA complexes. Third, VosA (or the VosA–VelB complex) positively controls the expression of trehalose synthase genes or negatively regulates the expression of genes associated with β-glucan biosynthesis.

Considering our results and those of previous reports [24,25,31], we confirmed the multifunctional role of VosA in *A*. *nidulans* ascospores (Figure 6). First, VosA plays a crucial role in spore viability during ascospore maturation. Second, VosA positively regulates trehalose biosynthesis by controlling the expression of trehalose synthase genes *tpsA* and *orlA*. It is unclear whether VosA interacts with VelB in ascospores. However, previous studies speculate that a VosA–VelB complex can bind to the promoter regions of certain genes and affect their expression [27]. Third, VosA controls the expression of secondary metabolite biosynthesis genes. Although it appears that VosA affects the production of certain secondary metabolites in ascospores, the molecular mechanisms are not clear. We speculate two possible ways: Either VosA directly or indirectly controls mRNA expression of secondary metabolite gene clusters, or deletion of *vosA* causes an increase in the number of VelB–VeA–LaeA complexes, which increases mRNA expression of secondary metabolite gene clusters. Further study will provide a better understanding of the molecular meFchanisms of VosA function in *A*. *nidulans*. Taken together, these results support the conclusion that VosA is a key regulator in *Aspergillus* spores.

## Figures and Tables

**Table 1:** Oligonucleotides used in this study.

Name	$\mathrm{Sequence}(5^{\prime }\to 3^{\prime })$	Purpose
OHS044	GTAAGGATCTGTACGGCAAC	5′ *actin* RT_F
OHS045	AGATCCACATCTGTTGGAAG	3′ *actin* RT_R
OHS576	GGTTGAAGTCGTCGGTTGAG	5′ *tpsA* RT_F
OHS577	TGGAAACCGATGAGGTCACA	3′ *tpsA* RT_R
OHS616	CTCCTACTCGCGTCACTTCT	5′ *orlA* RT_F
OHS617	AGGAAAGACATCCACAGCCA	3′ *orlA* RT_R
OHS1119	GATTATTCGGCCCAGAGGGA	5′ *ccg9* RT_F
OHS1120	ATGGCTTTCCACGTATTGGC	3′ *ccg9* RT_R
OHS795	AGCATCGTGGAACGAAATGG	5′ *treA* RT_F
OHS796	GAACTGCTGGCGGAATTGAT	3′ *treA* RT_R
OHS599	GCGCGAAGAAGACTTCAAC	5′ *aflR* RT_F
OHS600	TGCAATAACTGCCGACGAC	3′ *aflR* RT_R
OHS602	CGCATCATCCTCACAAGTTC	5′ *stcU* RT_F
OHS603	TGACCGTGATCTTCTTGTCG	3′ *stcU* RT_R
OHS604	GCTACTGTTCCAGGCGACTA	5′ *stcE* RT_F
OHS605	CACAGCTCTCCATCTCGGTA	3′ *stcE* RT_R

**Table 2:** Secondary metabolite clusters affected by VosA in ascospores.

Secondary Metabolite Gene Cluster (Number of Genes in the Cluster)	Upregulated Genes in the Δ*vosA* Ascospores
Asperthecin (3)	*aptA*, *aptB*, *aptC*
Derivative of benzaldehyde 1 and F9775 hybrid cluster 1 (9)	*dbaA*, *dbaB*, *dbaC*, *dbaE*, *dbaF*, *dbaH*, *dbaI*
Derivative of benzaldehyde 2 and F9775 hybrid cluster 2 (3)	*orsA*, *orsB*, *orsC*
Microperfuranone cluster (3)	*CYP620D1*, *AN3394*, *mica*
Monodictyphenone cluster (12)	*mdpA*, *mdpB*, *mdpD*, *mdpE*, *mdpF*, *mdpH*, *mdpI*, *mdpJ*, *mdpK*, *mdpL*,
Sterigmatocystin cluster (24)	*stcW*, *stcV*, *stcU*, *stcT*, *stcS*, *stcQ*, *stcO*, *stcN*, *stcI*, *stcF*, *aflR*, *stcE*, *stcC*, *stcB*, *stcA*, *stcK*, *AN7817*, *AN7819*, *AN7822*, *AN11017*, *AN11021*
*pkdA* cluster (11)	*pkdA*, *AN0524*, *AN0525*, *AN0526*, *AN0527*, *AN0528*, *AN0529*, *AN0530*, *AN0531*, *AN0533*

## Supplementary Materials

The following are available online at https://www.mdpi.com/2073-4425/11/1/103/s1, Figure S1: Venn diagram showing VosA-dependent genes in conidia and ascospores. Figure S2: Role of VosA in ascospore germination. Table S1: Gene Ontology (GO) term enrichment analysis of differentially expressed genes in Δ*vosA* ascospores performed on the Aspergillus Genome Database (AspGD) site. Table S2: Gene Ontology (GO) term enrichment analysis of differentially expressed genes in Δ*vosA* ascospores performed on the FungiFun site. Table S3: Differentially expressed genes of 25 secondary metabolite clusters in WT and Δ*vosA* ascospores.
